# HDL-Cholesterol Subfraction Dimensional Distribution Is Associated with Cardiovascular Disease Risk and Is Predicted by Visceral Adiposity and Dietary Lipid Intake in Women

**DOI:** 10.3390/nu16101525

**Published:** 2024-05-18

**Authors:** Domenico Sergi, Juana Maria Sanz, Alessandro Trentini, Gloria Bonaccorsi, Sharon Angelini, Fabiola Castaldo, Sara Morrone, Riccardo Spaggiari, Carlo Cervellati, Angelina Passaro, MEDIA HDL Research Group

**Affiliations:** 1 Department of Translational Medicine, University of Ferrara, 44121 Ferrara, Italy; domenico.sergi@unife.it (D.S.); sharon.angelini@unife.it (S.A.); fabiola.castaldo@unife.it (F.C.); sara.morrone@unife.it (S.M.); riccardo.spaggiari@edu.unife.it (R.S.); carlo.cervellati@unife.it (C.C.); angelina.passaro@unife.it (A.P.); 2 Department of Chemical, Pharmaceutical and Agricultural Sciences, University of Ferrara, 44121 Ferrara, Italy; 3 Department of Environmental and Prevention Sciences, University of Ferrara, 44121 Ferrara, Italy; trnlsn@unife.it; 4 Department of Translational Medicine, Menopause and Osteoporosis Center, University Center for Studies on Gender Medicine, 44121 Ferrara, Italy; gloria.bonaccorsi@unife.it

**Keywords:** cardiovascular disease, HDL-cholesterol, HDL subfractions, visceral adiposity, dietary lipids

## Abstract

HDL-cholesterol quality, including cholesterol distribution in HDL subfractions, is emerging as a key discriminant in dictating the effects of these lipoproteins on cardiovascular health. This study aims at elucidating the relationship between cholesterol distribution in HDL subfractions and CVD risk factors as well as diet quality and energy density in a population of pre- and postmenopausal women. Seventy-two women aged 52 ± 6 years were characterized metabolically and anthropometrically. Serum HDL-C subfractions were quantified using the Lipoprint HDL System. Cholesterol distribution in large HDL subfractions was lower in overweight individuals and study participants with moderate to high estimated CVD risk, hypertension, or insulin resistance. Cholesterol distribution in large, as opposed to small, HDL subfractions correlated negatively with insulin resistance, circulating triglycerides, and visceral adipose tissue (VAT). VAT was an independent positive and negative predictor of cholesterol distribution in large and small HDL subfractions, respectively. Furthermore, an increase in energy intake could predict a decrease in cholesterol levels in large HDL subfractions while lipid intake positively predicted cholesterol levels in small HDL subfractions. Cholesterol distribution in HDL subfractions may represent an additional player in shaping CVD risk and a novel potential mediator of the effect of diet on cardiovascular health.

## 1. Introduction

Cardiovascular disease (CVD) represents the primary cause of death globally [1], with its incidence continuing to rise in parallel with the upsurge of obesity and type 2 diabetes mellitus (T2DM) [2,3]. In this context, the metabolic aberrations driven by obesity and T2DM have a pivotal role in disrupting the homeostasis of the circulating lipid profile which, in turn, is strongly associated with CVD. In particular, a rise in circulating triglycerides along with LDL-cholesterol, particularly small-dense-LDL-cholesterol, and a decrease in HDL-cholesterol increases CVD risk [4]. While HDL-cholesterol has been traditionally deemed to be cardio protective, the relationship between HDL-cholesterol and cardiovascular mortality is U-shaped, with both low and high circulating HDL-cholesterol levels being associated with CVD and mortality [5,6,7]. Thus, the impact of HDL-cholesterol on cardiovascular health is not only dictated by its circulating levels, but also by the functionality of HDL particles, namely, their antioxidant, anti-inflammatory, and cholesterol efflux capacity, which are crucial for HDL lipoproteins to elicit their cardioprotective effects [8,9]. In line with this, an impairment in HDL-antioxidant, anti-inflammatory, and cholesterol efflux capacity is intimately linked with CVD [10,11,12,13,14,15]. In addition to HDL functionality, HDL-cholesterol distribution is also emerging as an important player in dictating the effect of HDL lipoproteins on cardiovascular health [16,17]. In particular, while an increase in cholesterol distribution in large HDL subfractions has been associated with improved cardiovascular health, the opposite is true for small HDL subfractions [18,19,20]. Indeed, small HDL subfractions have also been shown to be augmented in obese, type 2 diabetic, and insulin resistant individuals who typically harbor an increased CVD risk [21,22,23]. 

With regard to the modifiable lifestyle factors, diet is central in shaping cardio-metabolic health, with the consumption of highly processed foods promoting the development of obesity and its comorbidities [24,25], which, in turn, increase CVD risk [26]. In this regard, maintaining a positive energy balance, in which energy intake is higher than energy expenditure, is pivotal in fostering these metabolic aberrations by promoting adiposity gain, particularly at the central level [27,28]. However, the relationship between diet and cardiometabolic health is not only dependent upon the amount of energy introduced through the diet, but also on the quality of the nutrients consumed [29]. In particular, the overconsumption of long-chain saturated fatty acids, particularly as part of highly processed foods [30], has a key role in this context by promoting inflammatory responses [31] and insulin resistance [32,33], as well as increasing circulating LDL-cholesterol [34], despite not affecting or even increasing HDL-cholesterol levels [35,36]. On the contrary, the monounsaturated fatty acids and omega-3 fatty acids have been shown to elicit beneficial effects on cardiometabolic health. Indeed, oleic acid, a monounsaturated fatty acid, has been reported to induce the production and release of large chylomicrons and increase postprandial triglyceride clearance, promoting a shift from small dense LDL to large buoyant LDL lipoproteins [37]. At the same time, the consumption of the omega-3 fatty acids, eicosatetraenoic acid (EPA) and docosahexaenoic acid (DHA), are known to promote a cardioprotective circulating lipid profile characterized by a decrease in circulating triglycerides [38], LDL, and an increase in HDL-cholesterol [39]. 

Nevertheless, despite anthropometric and metabolic factors, as well as diet quality and energy density, all playing a role in the development and progression of CVD, their relationship with HDL-cholesterol subfraction dimensional distribution remains to be fully elucidated. Thus, this study aims to identify the relationship between the HDL-cholesterol subfraction dimensional distribution and anthropometric, metabolic, as well as nutritional risk factors for CVD in a population of fertile and postmenopausal women.

## 2. Materials and Methods

### 2.1. Study Cohort

In this mono-centric cross-sectional study, 72 Caucasian women aged 41–67 years (52 ± 6 years) were enrolled from volunteers attending the Menopause and Osteoporosis Centre of the University of Ferrara (Italy). Menopausal status was defined according to the Stages of Reproductive Aging Workshop (STRAW) [40]. Exclusion criteria were the presence of chronic diseases (diabetes, thrombo-embolism, neurodegenerative diseases, significant systemic infections, autoimmune diseases, malignant neoplastic diseases, or renal failure); menopause hormonal therapy (estrogen or estrogen-progestin) in the 6 months prior to the enrollment into the study; frequent use (on average, more than twice a week) of vitamin supplements or medications (anti-inflammatory, analgesic, anti-allergic, antidepressant); habitual consumption of alcoholic beverages (>30 g of alcohol/day); or a current smoking habit.

Study participants underwent anamnestic and nutritional interviews, anthropometric measurements, and fasting blood sampling. The study was carried out in accordance with the Declaration of Helsinki (World Medical Association, http://www.wma.net) and approved by the ethics committee of the University-Hospital of Ferrara (207/2019/Sper/UniFe, approved on 20 May 2019). Each subject signed an informed consent form prior to the inclusion in the research protocol.

### 2.2. Dietary Assessment

Participants were interviewed by clinical dietitians about food and drinks consumed in the 24 h prior to the interview (24 h recall) [41]. The interview was repeated after two months by the same member of staff. Total energy, macro as well as micronutrient intake, computed using Winfood^®^ PRO 3.3 (Medimatica Surl, Teramo, Italy), were the average of the values obtained from the two 24 h recalls.

### 2.3. Biochemical Analysis

Fasting blood samples were centrifuged at 1600× *g* for 15 min and serum or plasma isolated, aliquoted, and stored at −80 °C until use. Total cholesterol, HDL cholesterol, triglycerides, glucose, and insulin were assayed in serum using standard enzymatic colorimetric methods (Beckman Coulter, Brea, CA, USA). LDL cholesterol was computed by the Friedewald’s formula [42]. Insulin resistance was assessed using the homeostasis model assessment index (HOMA-IR), which was calculated as follows:

$$\mathrm{HOMA-IR}\;index=\frac{glucose\left(\frac{mmol}{L}\right)\cdot insulin(\frac{mU}{L})}{22.5}$$

### 2.4. Assessment of Anthropometric Indexes of Adiposity, Body Composition Using DXA, and Estimated CVD Risk

Body weight and height were measured by trained personnel, and BMI was computed thereafter. Waist circumference was measured around the smallest circumference between the lowest rib and iliac crest. The evaluation of densitometric parameters were performed using DXA (Hologic Discovery; software version APEX 3.3.0.1, Bedford MA, USA). The instrument’s software provided estimates of absolute fat free mass (FFM), fat mass (FM), and bone mineral mass (grams) for the total body and for standard body areas [43]. Cardiovascular risk was estimated using the Systematic COronary Risk Evaluation (SCORE) 2 algorithm (low risk < 1; moderate risk between ≥1 and <5; high between ≥5 and <10) [44].

### 2.5. Characterization of Cholesterol Distribution in HDL Subfractions

The quantification of cholesterol distribution in HDL subfractions was performed using the Lipoprint System (Quantimetrix Corporation, Redondo Beach, CA, USA) [18,45]. Briefly, Sudan Black pre-stained lipoprotein subfractions were separated on the basis of their size using electrophoresis on polyacrylamide gel. Successively, gels were scanned and analyzed using Lipoprint software, to determine cholesterol concentration in 10 subfractions (HDL1–HDL10). For some analyses, these subfractions were grouped into 3 subclasses: HDL1–3, large (l-HDL); HDL4–7, intermediate (m-HDL); and HDL8–10, small (s-HDL).

### 2.6. Statistical Analysis

Continuous variables were expressed as mean ± standard deviation (SD) and median (interquartile range). The distribution of continuous variables was evaluated using the Shapiro–Wilk test. Differences between two groups of normally distributed variables were assessed using Student *t*-test, whereas One Way ANOVA followed by a Bonferroni post hoc test was used when comparing three groups. Differences between two or three groups of not normally distributed variables were identified using Mann–Whitney or Kruskal–Wallis tests, respectively. Correlations between cholesterol distribution in HDL subfractions and clinical and nutritional parameters were identified using Spearman’s rank test. 

Forward stepwise multiple regression analysis was performed to reveal the independent predictors of cholesterol distribution in large and small HDL subfractions. In this case, s-HDL subfractions and other not normally distributed parameters were log-transformed. Data analysis was performed using SPSS Statistics for Windows, version 29.0 (SPSS, Inc., Chicago, IL, USA), and a *p* < 0.05 was considered as statistically significant.

## 3. Results

### 3.1. Characteristics of the Study Cohort

The characteristics of the 72 women included in this study are reported in Table 1. These include the metabolic and anthropometric profile of study participants, as well as the number of individuals affected by hypertension (n = 9, 12.5%), obesity (n = 9, 12.5%), and high estimated CVD risk according to SCORE2 (n = 3, 4.2%) (Table 1).

### 3.2. HDL-C Subfraction Distribution in Relation CVD Risk Factors

**Figure 1:**
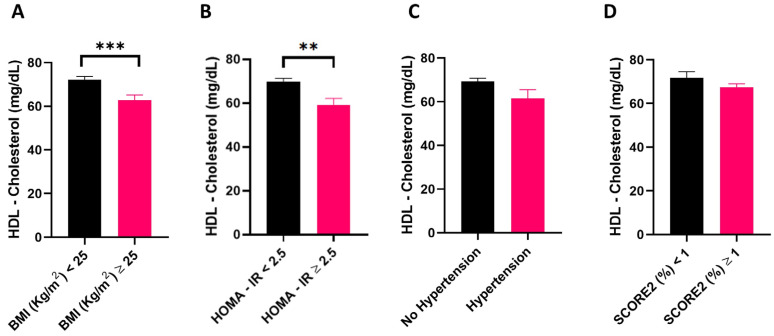
HDL-cholesterol circulating levels in overweight, hypertensive, insulin resistant, and individuals with moderate to high estimated CVD risk and their respective controls. Comparison of HDL-cholesterol between: normal weight (BMI < 25) (n = 43) versus overweight individuals (BMI ≥ 25) (n = 29) (**A**); individuals with HOMA-IR < 2.5; n = 62) versus insulin resistant individuals with HOMA-IR ≥ 2.5 (n = 10) (**B**); normotensive (n = 62) versus hypertensive individuals (n = 10) (**C**); individuals with low estimated CVD risk (n = 16) versus individuals with high to moderate CVD risk (n = 56) (**D**). Data are reported as mean ± standard error of the mean, and are analyzed using Student’s *t*-test. ** *p*-value < 0.01; *** *p*-value < 0.001. BMI, body mass index; HOMA-IR, homeostatic model assessment for insulin resistance.

**Figure 2:**
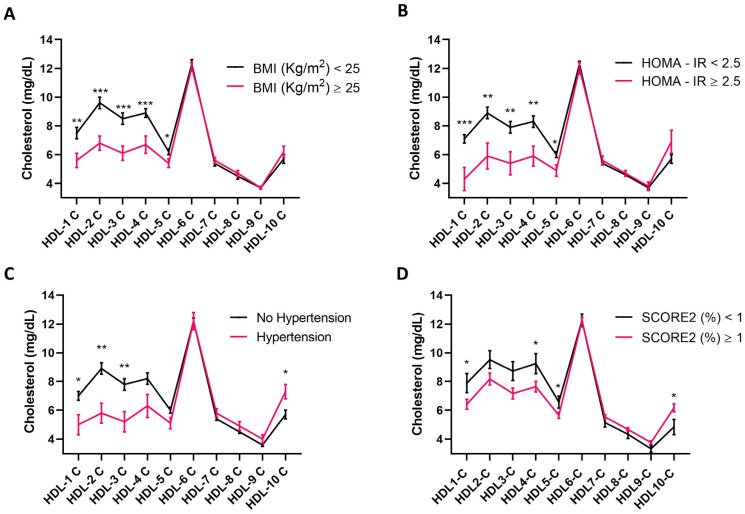
Distribution of cholesterol in HDL subfractions in overweight, hypertensive, insulin resistant, and individuals with moderate to high estimated CVD risk, and their respective controls. HDL-cholesterol subfraction dimensional distribution in normal weight (BMI < 25) (n = 43) versus overweight individuals (BMI ≥ 25) (n = 29) (**A**); individuals with HOMA-IR < 2.5; (n = 62) versus insulin resistant individuals with HOMA- IR ≥ 2.5 (n = 10) (**B**); normotensive (n = 62) versus hypertensive individuals (n = 10) (**C**); individuals with low estimated CVD risk (n = 16) versus individuals with high to moderate CVD risk (n = 56) (**D**). Data are reported as mean ± standard error of mean and are analyzed using Student’s *t*-test. * *p*-value < 0.05; ** *p*-value < 0.01; *** *p*-value < 0.001. BMI, body mass index; HOMA-IR, homeostatic model assessment for insulin resistance.

Before evaluating the relationship between cholesterol distribution in HDL subfractions and CVD risk factors, it was investigated whether HDL-cholesterol circulating levels differed in individuals who were overweight or who had hypertension and insulin resistance, all CVD risk factors [26,46,47], and they were stratified according to their estimated CVD risk assessed using SCORE2 [44]. HDL-cholesterol levels were lower in overweight (*p* = 0.001) (Figure 1A) and insulin-resistant individuals (*p* = 0.009) (Figure 1B) relative to their respective controls. The circulating concentration of HDL-cholesterol only tended to be significantly lower in hypertensive compared to normotensive study participants (*p* = 0.068) (Figure 1C), but it did not differ when comparing study participants according to their estimated CVD risk (*p* > 0.05) (Figure 1D). Considering an increase in cholesterol distribution in small HDL subfractions being observed in individuals harboring a higher CVD risk [18], it was next investigated whether this held true in the present study cohort. This observation was confirmed in the individuals who took part in this study, with overweight and obese individuals displaying a decrease in cholesterol distribution in HDL subfractions 1 to 5 compared to individuals with a BMI < 25 (Figure 2A). Similarly, individuals with hypertension had lower cholesterol levels in HDL subfractions 1, 2, and 3, and an increase in the small HDL-10 subfraction relative to their normotensive counterparts (Figure 2B). To a similar extent, individuals displaying insulin resistance, as indicated by a HOMA-IR ≥ 2.5, presented lower levels of cholesterol distribution in HDL subfraction 1 to 4 compared to insulin-sensitive controls (Figure 2C). Finally, cholesterol distribution in HDL subfractions 1, 4, and 5 was higher in individuals with a low, compared to those with a moderate to high, estimated CVD risk, whereas the opposite was true for HDL subfractions 10 (Figure 2D).

To further dissect the relationship between HDL-cholesterol subfraction dimensional distribution and CVD risk factors, it was evaluated how HDL-cholesterol subfraction dimensional distribution and HDL-cholesterol correlated with parameters known to affect cardiovascular risk. In this regard, the distribution of cholesterol in large HDL subfractions correlated negatively with BMI (*p* < 0.001), systolic blood pressure (SBP) (*p* = 0.015), visceral adipose tissue (VAT) (*p* < 0.001), fat mass (*p* < 0.001), circulating triglycerides (*p* < 0.001), APOB 100 (*p* = 0.012), insulinemia (*p* < 0.001), and HOMA-IR (*p* < 0.001). On the contrary, the levels of cholesterol in large HDL subfractions correlated positively with HDL-cholesterol (*p* < 0.001) and APO A1 (*p* < 0.001) (Table 2). Similarly to large HDL subfractions, the distribution of cholesterol in medium HDL subfractions correlated negatively with BMI (*p* = 0.039), VAT (*p* = 0.039), and circulating triglycerides (*p* = 0.001), as well as APOB 100 (*p* = 0.012), and correlated positively with HDL-cholesterol (*p* < 0.001) and APO A1 (*p* < 0.001) (Table 2). An opposite trend was observed for cholesterol distribution in small HDL subfractions which correlated positively with age (*p* = 0.032), BMI (*p* = 0.014), VAT (*p* < 0.001), fat mass (*p* = 0.007), total cholesterol (*p* < 0.001), circulating triglycerides (*p* < 0.001), LDL-cholesterol, APO B100 (*p* = 0.001), insulinemia (*p* = 0.008), and HOMA-IR (*p* = 0.010) (Table 2). With regard to HDL-cholesterol, it followed the same correlation pattern as the cholesterol distribution in large HDL subfractions (Table 2). 

### 3.3. Anthropometric Parameters as Predictors of HDL-Cholesterol Subfraction Dimensional Distribution

After confirming the relationship between cholesterol distribution in HDL subfractions and metabolic as well as anthropometric risk factors for CVD, it was explored whether the latter could serve as predictors of cholesterol distribution in both large and small HDL subfractions. In this regard, only VAT emerged as a predictor of cholesterol distribution in large HDL subfractions (Table 3A, *p* < 0.001). In particular, an increase in VAT was associated with a decrease in cholesterol distribution within large HDL subfractions. 

On the contrary, an increase in VAT was predictive of an increase in cholesterol abundance in small HDL subfractions, with this relationship being enhanced by fat mass (*p* = 0.040), as demonstrated in model 2 (Table 3B).

### 3.4. The Relationship between Diet and HDL-Cholesterol Subfraction Dimensional Distribution

Diet quality and energy density are key discriminants in modulating cardiometabolic health and, as a consequence, CVD risk [37,41,48]. In light of this, it was assessed whether nutritional factors, besides anthropometric and metabolic parameters, would also be able to affect HDL-cholesterol subfraction dimensional distribution. Daily energy and nutrient intake are reported in Table 4 Similar to what was described for anthropometric and metabolic parameters, a relationship between the cholesterol distribution in HDL subfractions and nutrient intake was also observed (Table 5). Indeed, while cholesterol distribution in large HDL subfractions correlated negatively with total lipid intake (*p* = 0.008), the opposite occurred for small HDL-cholesterol subfractions (*p* = 0.002). This effect appeared to be fatty-acid dependent, at least for small HDL-cholesterol subfractions, with a positive relationship being detected between cholesterol distribution in small HDL subfractions and saturated (*p* = 0.028) as well as monounsaturated fatty acid (*p* = 0.046) intake (Table 5). On the contrary, the negative correlation between saturated fatty acid intake and cholesterol distribution in large HDL-subfractions was not significant (*p* = 0.103), while it tended towards significance for the intake of monounsaturated fatty acids (*p* = 0.063) (Table 5).

**Figure 3:**
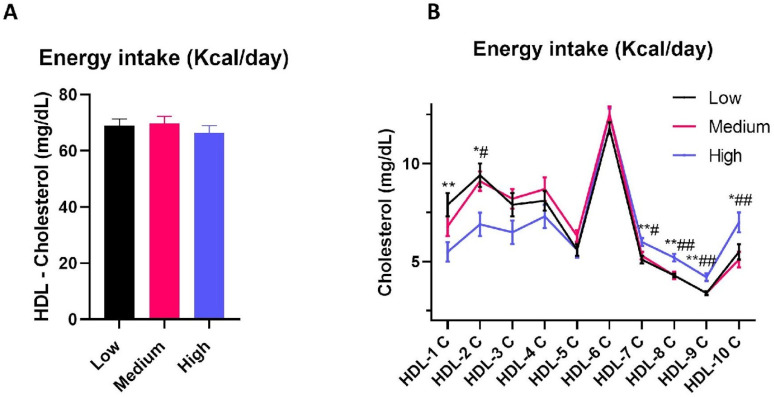
HDL cholesterol circulating levels and subfraction distribution between energy intake tertiles. HDL-cholesterol circulating levels (**A**) and distribution of cholesterol in HDL subfractions (**B**) in low energy intake (LOW), medium energy intake (MEDIUM), and high energy intake (HIGH) tertiles. Data are reported as mean ± standard error of the mean, and are analyzed using one-way ANOVA. *, low energy intake vs. high energy intake; #, medium energy intake vs. high energy intake. *, # *p*-value < 0.05; **, ## *p*-value < 0.01.

Furthermore, energy and lipid intake were also identified as predictors of HDL-cholesterol subfraction dimensional distribution. Particularly, an increase in energy intake was able to predict a decrease in cholesterol distribution in large HDL subfractions independently of saturated and monounsaturated fatty acid intake (Table 6A). In agreement with this, despite energy intake not affecting total HDL-cholesterol levels (Figure 3A), when stratifying the cohort according to energy intake, individuals in the highest tertile displayed an increase in cholesterol distribution in small HDL subfractions (HDL-8, -9, and -10) compared to those in the medium and low energy intake tertiles (Figure 3B). Furthermore, the study participants in the highest tertile of energy intake were characterized by a decrease in cholesterol levels in large HDL subfractions (HDL-1, -2, and -3) relative to individuals in the medium and low energy intake tertiles (Figure 3B). However, when considering cholesterol distribution in small HDL subfractions, an increase in total lipid consumption, rather than energy intake *per se*, was able to predict an increase in cholesterol levels in small HDL subfractions (Table 6B).

## 4. Discussion

The present study provides further support to the positive relationship between cholesterol distribution in small HDL subfractions and CVD risk, in line with previous reports [18,19,20]. In keeping with this, cholesterol distribution in small HDL subfractions displayed a positive relationship with known cardiovascular risk factors for CVD, whereas the opposite occurred for cholesterol levels in large HDL subfractions. Additionally, the data presented herein indicate that dietary lipid intake and energy density may impact upon cholesterol distribution in HDL subfractions. Particularly, an increase in energy intake emerged as being predictive for a decrease in cholesterol distributed within large HDL subfractions, whereas an increase in lipid intake was identified as a predictor of a rise in cholesterol within small HDL subfractions.

Despite HDL-cholesterol being deemed as cardioprotective, its circulating levels are not sufficient to explain its effects on cardiovascular health, especially considering that both low and high levels of plasma HDL-cholesterol are associated with CVD risk and mortality [5,6,7]. In this regard, cholesterol distribution in HDL subfractions may also be pivotal in shaping CVD risk. The data reported herein support the possibility that an increase in cholesterol distribution in large, as opposed to small, HDL-subfractions is associated with a decrease in CVD risk. This notion is corroborated by the fact that the distribution of cholesterol in HDL subfraction 1 is lower in individuals who are overweight, affected by hypertension and insulin resistance, and who have a moderate to high CVD risk score; however, an increase in cholesterol levels in small HDL subfractions are a peculiar characteristic of individuals affected by hypertension and who have increased CVD risk estimated using SCORE2, particularly when considering cholesterol distribution in HDL subfraction 10. Additionally, while cholesterol distribution in large HDL subfractions correlated negatively with known risk factors for CVD disease, the opposite was true for the amount of cholesterol distributed in small HDL subfractions. This finding is in agreement with previous reports, indicating that an increase in cholesterol distribution in small, as opposed to large, HDL subfractions is associated with increased CVD risk [18,49], and is observed in individuals affected by Familial Hypercholesterolemia characterized by early CVD [45]. Despite the observed relationship between cholesterol distribution in HDL subfractions and established CVD risk factors, it is not possible to infer from this or prior studies [49,50] whether an increase in cholesterol distribution within large HDL and a decrease in its levels within small HDL subfractions provide cardioprotective effects. Indeed, it remains to be elucidated whether the shift in cholesterol distribution in small HDL subfractions represents a compensatory mechanism driven by an upregulation of nascent HDL synthesis in order to mitigate the CVD risk or if, instead, this may be due to an increase in small HDL at the end of their life cycle. 

Therefore, the decrease in cholesterol distribution in large HDL subfractions may represent an adaptive response to the metabolic aberrations associated with body weight gain, central adiposity, and high LDL-cholesterol levels. In particular, it may be a consequence of an increased activity of cholesterol ester transfer protein (CETP), as observed in obese individuals [51], which mediates the exchange of triglycerides and cholesterol esters between triglyceride rich and LDL, as well as HDL lipoproteins [52]. This, in turn, results in an enrichment of HDL lipoproteins with triglycerides, which are readily catabolized and cleared with a consequent decrease in large and a concomitant increase in small HDL subfractions, as suggested by the data reported herein. Furthermore, it remains to elucidate the characteristics of these small HDL subfractions, and particularly if they lose their antioxidant and anti-inflammatory properties due to changes in the amount and activity of proteins like Paraoxonase or Myeloperoxidase in their proteome [9]. These metabolic irregularities may be a direct consequence of increased visceral adiposity which, in turn, is a key driver of insulin resistance [53,54,55]. In this regard, increased free fatty acid release from dysfunctional insulin-resistant visceral adipose tissue depots provide the substrates in order to increase triglyceride synthesis within the liver leading to a subsequent increase in very low-density lipoproteins (VLDL) export. These lipoproteins, in turn, supply triglycerides to HDL lipoproteins in exchange for cholesterol esters, with a consequent decrease in cholesterol-rich HDL2 [56,57]. This possibility is also supported by the fact that HDLs, enriched in triglycerides, undergo hydrolysis of their lipid component and lose APO A1. Indeed, as part of this study, it was found that APO A1 correlated positively only with large HDL subfractions. Furthermore, in agreement with this hypothesis, cholesterol distribution in large HDL subfractions correlated negatively with circulating triglycerides and APO B100, even though this apolipoprotein also characterized LDL-C apart from VLDL. Instead, the opposite is true for cholesterol distribution in small HDL subfractions. Thus, visceral adiposity and insulin resistance may be the responsible for the decrease in cholesterol distribution in large HDL subfractions in overweight individuals and those affected by hypertension, insulin resistance, and high LDL cholesterol levels. In support to this possibility, not only HOMA-IR and VAT correlated negatively with cholesterol distribution in large and positively in small HDL subfractions, but VAT was also able to predict cholesterol distribution in HDL subfractions. However, despite the fact that a decrease in cholesterol distribution in large HDL subfractions may potentially indicate a parallel decrease in large HDL subfractions, this cannot be inferred using the Lipoprint system. Indeed, this analytical technique does not directly quantify the amount of HDL subfractions; instead, it directly assesses the level of cholesterol in each on the ten HDL subfractions. In light of this, the data reported herein and in other studies [16,18,50] do not provide a direct readout of the amount of HDL subfractions, but rather the amount of cholesterol they carry. However, changes in cholesterol distribution in HDL subfractions may still represent an additional biomarker of cardiovascular risk.

Diet is a key player is shaping cardiometabolic health [37,41,48], as confirmed by the relationship between dietary parameters and cholesterol distribution in HDL subfractions reported herein. In particular, individuals in the highest tertile for energy intake also displayed an increase in cholesterol distribution in small, and a decrease in large, HDL subfractions, respectively. Additionally, an increase in energy intake was able to predict a decrease in cholesterol distribution in large HDL subfractions. These finding are in agreement with the fact that an energy-restricted low-carbohydrate dietary intervention aimed at eliciting body weight loss was able to increase large HDL subfractions [58], whereas an increase in adiposity, particularly central adiposity, led to a decrease in cholesterol distribution in large HDL subfractions [50]. An increase in energy intake, in the absence of a concomitant increase in energy expenditure, shifted the energy balance toward the positive, promoting body weight gain. This is in line with the data reported herein, with overweight individuals displaying a decrease in cholesterol abundance in large HDL subfractions. 

Despite energy intake emerging as a key driver of HDL-cholesterol subfraction dimensional distribution, this effect appears to be nutrient specific. Indeed, while the intake of carbohydrates and proteins did not correlate with the distribution of cholesterol in HDL subfractions, total lipid intake correlated positively with cholesterol distribution in small, and negatively in large, HDL subfractions. However, this effect, rather than being dependent upon cholesterol intake, appears to be driven by the intake of dietary fatty acids. Indeed, saturated and monounsaturated fatty acids correlated positively with cholesterol distribution in small HDL subfractions. The fact that both monounsaturated and saturated fatty acid intake are related to cholesterol distribution suggests that this the relationship between lipid intake and the levels of cholesterol in HDL subfractions may not be fatty acid specific, and may depend upon the overall lipid intake. This possibility is supported by the fact that lipid intake, after adjusting for individual fatty acid groups, was able to predict cholesterol distribution in small HDL subfractions independently from energy intake. Nevertheless, polyunsaturated fatty acids did not show any correlation with cholesterol distribution in HDL subfractions. This suggests that the impact of lipid intake on cholesterol distribution may be driven by the sum of monounsaturated and saturated fatty acids, rather than polyunsaturated fatty acids themselves. Saturated fatty acids, and particularly long-chain saturated fatty acids, are considered to be deleterious for cardiovascular health [59], whereas the opposite is true for monounsaturated fatty acids [37]. However, the fact that these fatty acids have the same relationship with cholesterol distribution in HDL subfractions is in contrast with the notion that a decrease in cholesterol distribution in large, and an increase in small, HDL subfractions is associated with CVD risk [18]. The reason for this apparent discrepancy remains to be elucidated. Indeed, while there are reports describing the impact of dietary fatty acids on LDL cholesterol subfractions [17,37,60], to the best of our knowledge, this is the first report describing the relationship between fatty acid quality and HDL-cholesterol subfraction distribution. Nevertheless, these fatty acids may differently modulate HDL lipoprotein functionality [61], independently of cholesterol distribution, and therefore still exert opposite effects on cardiovascular health. 

This study is not without limitations. First, its retrospective nature and the lack of information about pharmacological or dietary intervention prevented us from directly evaluating how the modulation of CVD risk factors reflects upon HDL-cholesterol subfraction dimensional distribution. For this, a longitudinal study setup would be more appropriate. Second, dietary data are derived from 24 h recalls which, despite being a validated method, do not provide information to directly infer a cause–effect relationship between energy, as well as nutrient, intake and cholesterol distribution in HDL subfractions. Finally, the sample size of the cohort represents an additional limitation of the study which, however, was mitigated by the fact that the study’s participants underwent an extensive metabolic characterization. Another aspect that should not be overlooked is related to the fact that the present study only includes female participants. Despite this, the data presented herein are in line with previous reports that included both males and females [18,19,20]. Nevertheless, this study, to the best of our knowledge, is the first to provide evidence of the relationship between dietary intake and cholesterol distribution in HDL subfractions which, instead, represents a strength of the present report.

## 5. Conclusions

In summary, this study sheds further light on the relationship between cholesterol distribution in different HDL subfractions and CVD risk. In particular, the data reported herein highlight the role of visceral adiposity, a key risk factor for CVD, as a predictor of cholesterol distribution in large as well as small HDL subfractions, albeit the present findings are limited to females. Thus, cholesterol distribution in HDL subfractions may represent an additional player in shaping cardiovascular health and a novel putative biomarker of CVD risk. Additionally, given the role of diet, and particularly its energy density, in predicting cholesterol abundance in large HDL subfractions, cholesterol distribution may be an additional factor involved in mediating the effect of diet and cardiovascular health.

## Figures and Tables

**Table 1:** General characteristics of the study population.

	Median (IQR)
Age (years)	53 (47–57)
BMI (Kg/m^2^)	24.3 (22.6–27.6)
DBP (mmHg)	70 (65–77)
SBP (mmHg)	112 (102–125)
Fat Mass (kg)	26.0 (21.9–31.3)
Free Fat Mass (kg)	36.9 (33.7–41.1)
VAT (kg)	0.49 (0.29–0.67)
Triglycerides (mg/dL)	72.8 (61.0–100.9)
Total-C (mg/dL)	236.1 (215.1–262.3)
HDL-C (mg/dL)	69.9 (59.1–76.9)
LDL-C (mg/dL)	148.1 (132.3–174.4)
APO A1 (mg/dL)	178.5 (164.8–203.0)
APO B100 (mg/dL)	91.0 (81.0–103.5)
Glycemia (mg/dL)	95.6 (90.5–99.7)
Insulinemia (U/L)	4.9 (3.4–6.9)
HOMA-IR	1.1 (0.8–1.7)
	**n (%)**
Hypertension, n (%)	9 (12.5)
Obesity, n (%)	9 (12.5)
SCORE2 (low), n (%)	16 (22.2)
SCORE2 (moderate), n (%)	53 (73.6)
SCORE2 (high), n (%)	3 (4.2)

Data are expressed as median (interquartile range, IQR) or n (%). BMI, Body Mass Index; SBP, systolic blood pressure; DBP, diastolic blood pressure; Total-C, total cholesterol; HDL-C, high-density lipoprotein cholesterol; LDL-C: low-density lipoprotein cholesterol; HOMA-IR, Homeostatic Model Assessment for Insulin Resistance; SCORE2, algorithm to estimate 10-year risk of cardiovascular disease in Europe; n, number of subjects.

**Table 2:** Spearman’s rho correlation between HDL-C subclasses and clinical parameters.

	HDL-C (mg/dL)		l-HDL-C (mg/dL)		m-HDL-C (mg/dL)		s-HDL-C (mg/dL)	
	Rho	*p*-Value	Rho	*p*-Value	Rho	*p*-Value	Rho	*p*-Value
Age (years)	0.077	0.520	−0.038	0.750	0.038	0.753	0.253	**0.032**
BMI (Kg/m^2^)	−0.385	**0.001**	−0.541	**<0.001**	−0.244	**0.039**	0.288	**0.014**
SBP (mmHg)	−0.258	**0.029**	−0.285	**0.015**	−0.172	0.149	0.196	0.098
DBP (mmHg)	−0.175	0.143	−0.228	0.054	−0.069	0.563	0.158	0.184
VAT (kg)	−0.374	**0.001**	−0.607	**<0.001**	−0.243	**0.039**	0.477	**<0.001**
FM (kg)	−0.354	**0.002**	−0.536	**<0.001**	−0.202	0.089	0.317	**0.007**
FFM (kg)	−0.227	0.056	−0.161	0.178	−0.158	0.184	−0.066	0.583
Triglyceride (mg/dL)	−0.447	**<0.001**	−0.602	**<0.001**	−0.397	**0.001**	0.439	**<0.001**
Total-C (mg/dL)	0.174	0.143	0.016	0.897	0.110	0.357	0.411	**<0.001**
HDL-C (mg/dL)			0.816	**<0.001**	0.922	**<0.001**	0.019	0.875
LDL-C (mg/dL)	−0.081	0.497	−0.164	0.169	−0.133	0.266	0.350	**0.003**
APO A1 (mg/dL)	0.832	**<0.001**	0.559	**<0.001**	0.818	**<0.001**	0.211	0.077
APO B100 (mg/dL)	−0.218	0.070	−0.297	**0.012**	−0.244	**0.042**	0.389	**0.001**
Glycemia (mg/dL)	−0.051	0.672	−0.149	0.211	0.063	0.600	0.099	0.410
Insulin (mU/L)	−0.323	**0.006**	−0.481	**<0.001**	−0.138	0.247	0.308	**0.008**
HOMA-IR	−0.302	**0.010**	−0.466	**<0.001**	−0.114	0.339	0.301	**0.010**

Rho, Spearman’s rho correlation coefficient; BMI, Body Mass Index; SBP, systolic blood pressure; DBP, diastolic blood pressure; VAT, visceral adipose tissue; FM, fat mass; FFM, Free Fat Mass; Total-C, total cholesterol; HDL-C, high-density lipoprotein cholesterol; LDL-C: low-density lipoprotein cholesterol; HOMA-IR, Homeostatic Model Assessment for Insulin Resistance. Significant correlations are reported in bold.

**Table 3:** Stepwise linear regression model indicating predictors of HDL-C subclasses: l-HDL-C (**A**) and log s-HDL-C (**B**).

**(A). l-HDL-C (mg/dL)**					
Model	R^2^	*p*-value model	Predictor	Unstandardized B coefficient	*p*-value variable
1	0.387	<0.001	log VAT	−20.916	<0.001
Model 1 excluded variables: age (years); log BMI (Kg/m^2^); log Fat Mass (Kg); log HOMA-IR; SBP (mmHg).					
**(B). log s-HDL-C (mg/dL)**					
Model	R^2^	*p*-value model	Predictor	Unstandardized B coefficient	*p*-value variable
1	0.215	<0.001	log VAT	0.222	<0.001
2	0.262	<0.001	log VAT	0.388	<0.001
			log Fat mass	−0.362	0.040
Model 1 excluded variables: age (years); log BMI (Kg/mq); log Fat mass; log HOMA-IR; SBP (mmHg).					
Model 2 excluded variables: age (years); log BMI (Kg/m^2^); log HOMA-IR; SBP (mmHg).					

BMI, Body Mass Index; SBP, systolic blood pressure; DBP, diastolic blood pressure; VAT, Visceral adipose tissue; FM, Fat mass; HOMA-IR, Homeostatic Model Assessment for Insulin Resistance. l-HDL-C, large HDL-cholesterol subfractions; s-HDL-C small HDL-cholesterol subfractions.

**Table 4:** Energy and nutrient intake of the study population.

	Daily Intake
	Median (IQR)
Calories (kcal)	1998.9 (1807.7–2223.9)
Protein (g)	87.5 (73.8–97.9)
Lipid (g)	89.5 (79.8–101.3)
Carbohydrates (g)	216.1 (186.2–253.8)
Total Fiber (g)	20.5 (15.7–23.0)
Cholesterol (mg)	215.0 (174.1–271.9)
SFA (g)	22.7 (18.0–28.5)
PUFA (g)	11.2 (9.1–13.3)
MUFA (g)	47.0 (39.9–56.6)

Data are expressed as median (interquartile range, IQR). SFA, Saturated Fatty Acid; PUFA, Polyunsaturated Fatty Acid; MUFA, Monounsaturated Fatty Acid.

**Table 5:** Spearman’s rho correlation between cholesterol distribution in HDL subfractions and dietary parameters.

	l-HDL-C (mg/dL)		m-HDL-C (mg/dL)		s-HDL-C (mg/dL)	
	Rho	*p*-Value	Rho	*p*-Value	Rho	*p*-Value
Calories (kcal/day)	−0.229	0.053	0.042	0.726	0.197	0.096
Protein (g/day)	−0.140	0.240	0.051	0.673	0.035	0.773
Lipid (g/day)	−0.309	**0.008**	0.111	0.352	0.357	**0.002**
Carbohydrates (g/day)	−0.150	0.209	−0.053	0.656	0.105	0.380
Total Fiber (g/day)	−0.015	0.902	−0.043	0.721	0.009	0.940
Cholesterol (mg/day)	0.060	0.616	0.207	0.081	−0.033	0.786
SFA (g/day)	−0.194	0.103	0.218	0.066	0.259	**0.028**
PUFA (g/day)	−0.117	0.328	0.036	0.761	0.177	0.137
MUFA (g/day)	−0.221	0.063	0.059	0.625	0.235	**0.046**

SFA, Saturated Fatty Acid; PUFA, Polyunsaturated Fatty Acid; MUFA, Monounsaturated Fatty Acid. Significant correlations are reported in bold.

**Table 6:** Stepwise linear regression model indicating predictors of l-HDL-C (**A**) and s-HDL-C (**B**) subclasses.

**(A). l-HDL-C (mg/dL)**					
Model	R^2^	*p*-value model	Predictor	Unstandardized B coefficient	*p*-value variable
1	0.081	0.015	Calories (kcal/day)	−0.007	0.015
Model 1 excluded variables: lipid (g/day); SFA (g/day); MUFA (g/day)					
**(B). log s-HDL-C (mg/dL)**					
Model	R^2^	*p*-value model	Predictor	Unstandardized B coefficient	*p*-value variable
1	0.098	0.007	Lipid (g/day)	0.002	0.007
Model 1 excluded variables: calories (kcal/day); SFA (g/day); MUFA (g/day)					

SFA, Saturated Fatty Acid; MUFA, Monounsaturated Fatty Acid.

## Data Availability

Data described in the manuscript will be made available from the corresponding author, Juana Maria Sanz (Department of Chemical, Pharmaceutical and Agricultural Sciences, University of Ferrara, Ferrara, Italy, juana.sanz@unife.it), upon reasonable request. In order for the data to be shared with interested parties, it will be required to sign a data access agreement.
